# Effect of Ozone Exposure on Cardiovascular and Cerebrovascular Disease Mortality in the Elderly

**DOI:** 10.3390/toxics13030184

**Published:** 2025-02-28

**Authors:** Tianyun Wang, Junlong Wang, Li Sun, Ye Deng, Yuting Xiang, Yuting Wang, Jiamei Chen, Wen Peng, Yuanyao Cui, Miao He

**Affiliations:** 1 Liaoning Key Laboratory of Environmental Health Damage Research and Assessment, Department of Environmental Physical Factors and Health, School of Public Health, Ministry of Education, China Medical University, Shenyang 110122, China; tianyun_wang0341@163.com (T.W.); dengye_dy@163.com (Y.D.); xiangyuting0130@163.com (Y.X.); wangyuting_0206@163.com (Y.W.); chenjiamei202@163.com (J.C.); pengwen@cmu.edu.cn (W.P.); cuiyuanyao@aliyun.com (Y.C.); 2 Liaoning Provincial Center for Disease Control and Prevention, Shenyang 110005, China; jundragon2001@163.com (J.W.); sl13940237963@163.com (L.S.); 3 Key Laboratory of Environmental Stress and Chronic Disease Control & Prevention, Ministry of Education, China Medical University, Shenyang 110122, China

**Keywords:** O_3_, PM_2.5_, joint effect, cardiovascular disease, cerebrovascular disease

## Abstract

Background: Ozone pollution has increased alongside China’s economic development, contributing to public health issues such as cardiovascular and cerebrovascular diseases. At present, the problem of an aging population is aggravated, which is worth more attention in terms of the health problems of elderly people. Methods: This study employed a distributional lag nonlinear model (DLNM) with Poisson regression to analyze the impact of ozone on cardiovascular and cerebrovascular disease mortality among the elderly in Shenyang, China, from 2014 to 2018. In addition, a time-series generalized additive regression model (GAM) was used to analyze the joint effect between PM_2.5_ and ozone. Results: We found a positive correlation between ozone and mortality from cardiovascular and cerebrovascular diseases in the elderly. The maximum relative risk (RR) of mortality from cardiovascular and cerebrovascular diseases for every 10 μg/m^3^ increase in ozone was 1.005 (95% CI: 1.002–1.008). Males (RR: 1.018, 95% CI: 1.007–1.030), individuals in unconventional marital status (RR: 1.024, 95% CI: 1.011–1.038), and outdoor workers (RR: 1.017, 95% CI: 1.002–1.031) were more vulnerable to ozone pollution. This study did not find significant differences in the impact of ozone pollution on cardiovascular and cerebrovascular disease mortality risks among different educational groups. Additionally, a joint effect between ozone and PM_2.5_ was observed. Conclusion: This study confirms that ozone exposure is positively associated with increased mortality from cardiovascular and cerebrovascular diseases. It emphasizes the joint effect of ozone and PM_2.5_ in exacerbating cardiovascular and cerebrovascular disease mortality.

## 1. Introduction

Ozone (O_3_) is a secondary pollutant formed through photochemical reactions involving nitrogen oxides (NO_x_) and volatile organic compounds (VOCs) under sunlight exposure [1]. As the continuous development of China’s economy, surface ozone pollution has intensified across various regions over the past two decades. Furthermore, near-surface ozone concentrations will continue to rise due to future climate change [2]. Rising O_3_ concentrations pose a serious public health threat [3]. Approximately 400,000 people worldwide died from O_3_ pollution in 2019, according to the 2019 Global Burden of Disease, Injury, and Risk Factors Study (GBD) [4]. Previous studies have shown a 46% increase in global O_3_-attributable mortality over a 20-year period [5]. Ozone exposure has been linked to various diseases, including respiratory diseases [6], cardiovascular diseases (CVD) [7,8], and cerebrovascular diseases (CEVD) [2,9]. Moreover, the population-weighted average O_3_ concentration in China is higher than in most other countries [10].

While numerous studies have investigated the effects of O_3_ on CVD and CEVD, the results remain inconsistent. Large cohort studies in the United States and Canada have found a positive correlation between increased O_3_ concentrations and CVD mortality [11,12]. However, a cohort study from the United Kingdom reported the opposite result [13]. Several studies have shown that O_3_ is positively associated with CVD mortality only during the warmer months [14,15]. Similarly, studies on CEVD and O_3_ are inconsistent. A study from Guangzhou found a positive correlation between O_3_ levels and the incidence of CEVD [16]. In contrast, another study from Taiwan concluded that O_3_ was negatively associated with CEVD incidence [17]. These discrepancies underscore the need for further investigation into the effects of O_3_ on CVD and CEVD mortality.

In addition to O_3_, fine particulate matter (PM_2.5_) has been widely recognized for its adverse effects on both CVD [18,19,20] and CEVD [17]. Although PM_2.5_ pollution has decreased in China in recent years [21], both O_3_ and PM_2.5_ remain major pollutants, often interacting and compounding their negative effects on human health [22]. Currently, few studies have been conducted on the joint effects of PM_2.5_ and O_3_. It is crucial to fully understand the joint effects of PM_2.5_ and O_3_ on CVD and CEVD.

Furthermore, China has become a rapidly aging society [23]. It is important to conduct studies in the elderly to reduce the burden of deaths caused by air pollution. This study aims to quantify the risks of O_3_ exposure on CVD and CEVD mortality in the elderly and examine whether these risks are modified by individual factors. Additionally, the study will investigate the joint effects of O_3_ and PM_2.5_ using both single-pollutant and two-pollutant models.

## 2. Materials and Methods

### 2.1. Study Area

**Figure 1:**
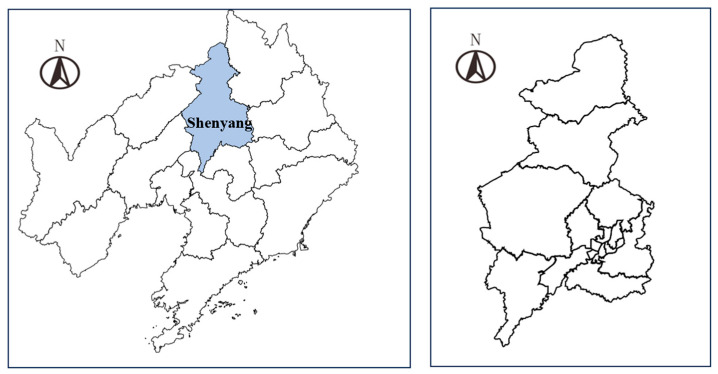
The map of Shenyang city, Liaoning province.

Shenyang, as the capital of Liaoning Province, is the center of transportation, economy, science and technology, commerce, and culture in Northeast China. It has a sub-humid temperate continental climate, with hot and humid summers and cold, dry winters. In Shenyang, the primary source of air pollution is soot, with PM_2.5_ being the main pollutant in winter and O_3_ the dominant pollutant in summer. In Shenyang, the majority of sulfur SO_2_ and soot emissions come from industrial sources, followed by residential emissions, with a small portion from vehicle emissions. For NO_x_ emissions, industrial sources remain the primary contributor, followed by vehicle emissions, with only a small fraction coming from residential emissions (Figure 1).

### 2.2. Data Collection

In this study, we collected data on deaths from 1 January 2014 to 31 December 2018 in Shenyang from the death registration system of the Liaoning Provincial Center for Disease Control and Prevention. The study focused on elderly individuals (aged ≥ 65) who died from cardiovascular disease (CVD; ICD-10 codes I05–I52) and cerebrovascular disease (CEVD; ICD-10 codes I60–I69). Participants were categorized based on gender, educational level, marital status, and occupation.

This study collected daily maximum 8-hour average concentrations of ozone during the study period from the Shenyang Municipal Environmental Protection Department (https://sthjj.shenyang.gov.cn/, accessed on: 25 Febuary 2025). In addition, we calculated daily 24-hour average concentration data, including PM_2.5_, SO_2_, NO_2,_ and CO.

Daily meteorological data, including average daily temperature (°C) and relative humidity (%), were provided by the meteorological monitoring site of the Liaoning Provincial Environmental Protection Department.

### 2.3. Statistical Analysis

This study used a distributed lag nonlinear model (DLNM) to analyze the lag–exposure–response relationship between ozone and CVD and CEVD deaths. In addition, this study used a generalized additive model (GAM) to analyze the interaction between O_3_ and PM_2.5_. Spearman was used for correlation analysis between pollutants and meteorological conditions.

#### 2.3.1. Distributed Lag Nonlinear Model (DLNM)

Since daily deaths from CVD and CEVD in the elderly are rare events, they followed a quasi-Poisson distribution. Therefore, DLNM with quasi-Poisson regression was employed to analyze the associations between O_3_ and mortality. Meteorological factors (temperature and relative humidity), other pollutants (PM_2.5_, NO_2_, SO_2_), and long-term trends were incorporated into the model to control for their potential impact on the outcome and to improve the accuracy of the model. The model is specified as follows:

*Yt*~Poisson (ut)

Log[E(Yt)] = β_0_ + β_1_cb.O_3t_ + β_2_cb.temp_t_ + ns(PM_2.5_,df) + ns(NO_2_,df) + ns(SO_2_,df) + ns(h,df) + ns(time,df) + Dow

Yt: The number of deaths due to cardiovascular and cerebrovascular disease on day t.
β_0_: The intercept.
β: The vector of coefficients.
cb.O_3t_: Cross-basis of O_3_ concentration at day t.
cb.temp_t_: Cross-basis of temperature at day t.
ns: Natural cubic spline.
df: Degree of freedom.
time: Time variable, to control long-term trend and seasonality.
dow: The day of week and public holidays are represented as categorical variables to control short-term fluctuations.

The degrees of freedom (df) for each variable were determined based on previous studies and the Akaike information criterion (AIC). Specifically, the df for daily average temperature was set to 5; for other pollutants (PM_2.5_, SO_2_, and NO_2_), it was set to 3; for humidity, it was set to 4; and for time, it was set to 7 per year.

Previous studies on the lag effect of air pollutants on deaths usually occur within 7 days [24,25]. In this study, single-day lag (lag0–lag7) and multi-day cumulative lag (lag00–lag07) were used to assess the lag-specific and cumulative effects of O_3_ on the deaths of cardiovascular and cerebrovascular diseases in the elderly. This study further explored the potential effects of gender, level of education, marital status, and season (warm season: April to October; cold season: November to March).

#### 2.3.2. Generalized Additive Model (GAM)

First, this study used a stratified analysis to assess the interaction between O_3_ and PM_2.5_. We stratified the daily concentrations of PM_2.5_ into two levels, low and high, according to the median of the study period.O_3_-8h is used as a continuous variable in the model to compare the risk of death from O_3_ at different stratification levels. The model is as follows:

Log[E(Yt)] = β_0_ + β(O_3_ × PM_2.5low_) + COVs

Log[E(Yt)] = β_0_ + β_1_(O_3_ × PM_2.5high_) + COVs

COVs: Covariates including time, mean temperature, relative humidity, weekday, public holidays, and the intercept.

Next, we used a two-pollutant model to analyze the joint effects of PM_2.5_ and O_3_, specified as follows:

Log[E(Yt)] = β_0_ + te(O_3_, PM_2.5_) + COVs

te (): Smoothing function that simultaneously fits the joint effect of the two variables.

All statistical analyses were performed using RStudio version 4.2.1, with the “mgcv”, “ggplot2”, and “dlnm” packages. The relative risks (RR) and 95% confidence intervals (95% CIs) for both individual and joint effects of PM_2.5_ and O_3_ are reported for every 10 μg/m^3^ increase in pollutant concentrations.

## 3. Results

### 3.1. Descriptive Statistics and Correlation Coefficients

As shown in Table 1, 117,604 people were included in this study, all of whom died of cardiovascular and cerebrovascular diseases from 2014 to 2018. The average age of the participants was 80.3 ± 7.85, of which 50.4% were male. Moreover, the participants were stratified by education, marital status, and occupation. Since the participants were all over 65 years old, the proportion of people with a junior high school and below level of education was high, forming 86.9% of the total.

Table 2 presents the average levels of temperature, relative humidity, and air pollution across different seasons during the study period. The O_3_ concentration in the warm season was significantly higher than in the cold season. The average O_3_ concentration in the warm season was 84.7 μg/m^3^, well above the WHO’s air quality guideline of 60 μg/m^3^.

Table 3 presents the correlation coefficients between air pollutants, temperature, and relative humidity (*p* < 0.01). PM_2.5_, PM_10_, SO_2_, NO_2_, and CO were positively correlated with each other and negatively correlated with temperature. The correlation between PM_2.5_ and PM_10_ is the strongest (r = 0.89), indicating a high degree of consistency in their concentration variations. O_3_-8 h was negatively correlated with other pollutants and positively correlated with temperature (r = 0.69).

### 3.2. The Association Between O_3_ and Mortality

Table 4 illustrates the relative risk (RR) (95% CI) of CVD and CEVD mortality for each 10 μg/m^3^ increase in O_3_ across different seasons. Model 1 did not adjust for other factors. Model 2 adjusted for temperature, humidity, week effects, and holiday effects. Model 3 incorporated PM_2.5_ based on Model 2. Model 4 adjusted SO_2_ and NO_2_ based on Model 3 to build the final model. Except for Model 1, the relative risk (RR) differences in the other three models were not significant, particularly in the warm season.

Table 5 summarizes the mortality risk for CVD and CEVD in the elderly in Shenyang for each 10 μg/m^3^ increase in O_3_ across a lag of 0–7 days. In the full season, the effect was highest at lag1 (RR = 1.005, 95% CI: 1.002–1.007). As for the cumulative lag, the maximum is observed at lag06, with a cumulative RR value of 1.009 (95% CI: 1.001–1.016). In the warm season, the effect was most significant at lag1 (RR = 1.007, 95% CI: 1.004–1.010). As for the cumulative lag, the effect of O_3_ increased slowly from lag01 to lag07, reaching a maximum at lag06, with a cumulative RR = 1.017 (95% CI: 1.009–1.026). The effect of O_3_ on the death risk of CVD and CEVD in the elderly was not statistically significant in the cold season. Therefore, further subgroup analyses were conducted for the warm season.

### 3.3. Subgroup Analysis of O_3_ Exposure

**Figure 2:**
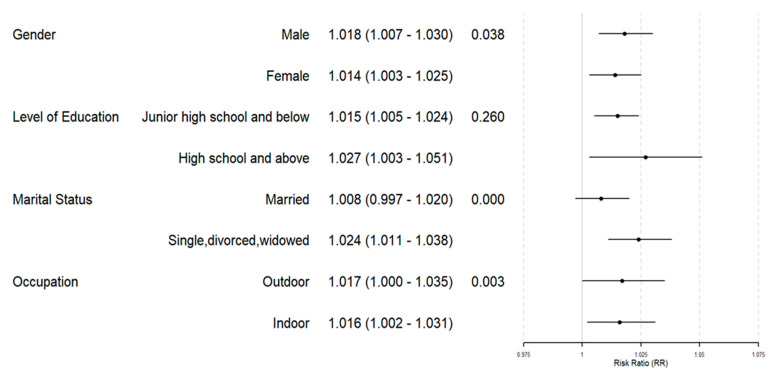
Associations of cardiovascular mortality with a 10 μg/m^3^ increase in warm-season O_3_ concentrations for different subgroups.

Figure 2 presents the RR (95% CI) for different subgroups in the warm season with each 10 μg/m^3^ increase in O_3_ concentration. It can be seen that the effect of O_3_ on CEVD and CVD mortality was greater in males than in females (*p* = 0.038). Single, divorced, and widowed individuals were more vulnerable to O_3_ exposure than married individuals, with a statistically significant difference between groups (*p* = 5.82 × 10^−11^). Additionally, outdoor workers had a significantly higher risk compared to indoor workers (*p* = 0.003).

**Figure 3:**
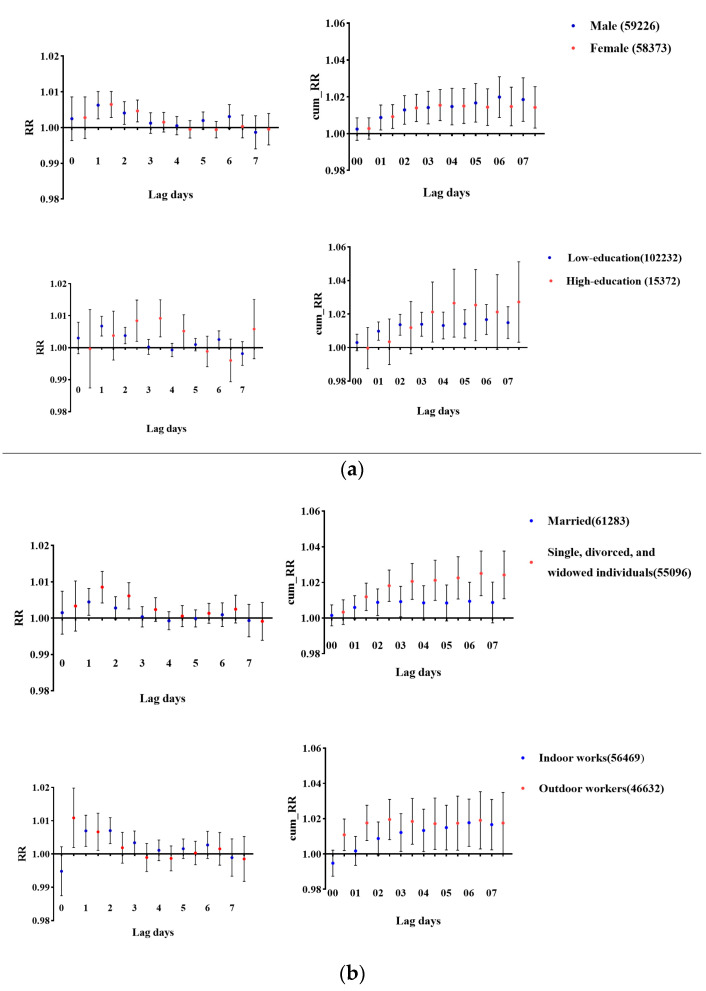
(**a**) The relative risk (lag0–7) and (**b**) the cumulative relative risk (lag00–07) per 10 μg/m^3^ of O_3_ elevation for a lag of 0–7 days in different subgroups.

Figure 3 shows the RR (95% CI) and cumulative RR (95% CI) for different subgroups at a lag of 0–7 days. Males exhibited the largest cumulative RR value at lag06. The RR was 1.020 (95% CI: 1.009–1.026) per 10 μg/m^3^ increase in O_3_. For females, the cumulative RR was 1.014 (95% CI: 1.006–1.023) per 10 μg/m^3^ rise in O_3_ at lag03. In the low-education group, the cumulative RR was statistically significant for lag01 through lag06 with the highest cumulative effect at lag06 (RR = 1.017, 95% CI: 1.008–1.026). The impact of O_3_ on married individuals was not significant. It was an upward trend in the cumulative RR for single, divorced, and widowed individuals. Among outdoor workers, the cumulative RR peaked at lag02 (RR = 1.020, 95% CI: 1.008–1.030) but showed a declining trend after lag02.

**Figure 4:**
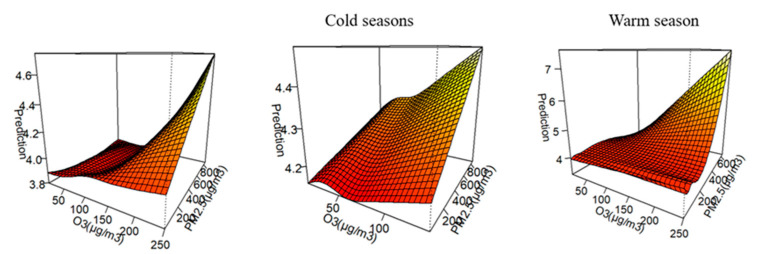
Bivariate response of PM_2.5_ and O_3_ to CEVD and CVD deaths in the elderly in Shenyang.

As shown in Figure 4, the risk of death from CEVD and CVD followed an increasing trend when both PM_2.5_ and O_3_ rose. Additionally, it can be observed that in the cold season, O_3_ concentrations are lower while PM_2.5_ concentrations are higher. In the warm season, the mortality rate of cardiovascular and cerebrovascular diseases in the elderly is more strongly influenced by the combined effect of O_3_ and PM_2.5_.

### 3.4. Joint Effect Between O_3_ and PM_2.5_

Table 6 describes the joint effect of O_3_ and PM_2.5_ on CEVD and CVD mortality. The RR for CEVD and CVD mortality at high PM_2.5_ were higher than those at low PM_2.5_ in both the single-pollutant model and two-pollutant model. The RR in the two-pollutant model is slightly more attenuated than in the single-pollutant model.

## 4. Discussion

This paper examined the relationship between O_3_ and the CVD and CEVD mortality in the elderly. We found a positive association between O_3_ concentration and the mortality risk from CVD and CEVD (RR: 1.007, 95% CI: 1.000–1.015). This association was stronger in the warm season compared to the cold season (RR: 1.01, 95% CI: 1.007–1.025). Males, individuals with low education, individuals with non-conventional marital status, and outdoor workers are more sensitive to O_3_ pollution. Additionally, we observed a synergistic effect of O_3_ and PM_2.5_ on CVD and CEVD mortality.

### 4.1. Effect of Ozone on CVD and CEVD

It indicated a significant relationship between O_3_ exposure and elevated mortality risk for CVD and CEVD in the elderly (RR: 1.007, 95% CI: 1.000–1.015). Previous studies have supported this observation [26,27,28]. Research on the relationship between O_3_ pollution and CEVD mortality has yielded inconsistent results. Studies from Taipei (OR: 1.031, 95% CI: 1.008–1.054) and Shenyang (ERR: 1.02%, 95% CI: 0.63–1.4%) have shown a positive correlation between the onset of CEVD and O_3_ exposure [29,30]. Furthermore, a nationwide study in China found that O_3_ is positively associated with the risk of CEVD hospitalization. The excess risk (ER) of hospitalization for CEVD per 10 μg/m^3^ increase in ozone concentration was 0.29% (95% CI: 0.18–0.40%) [31]. Research in Wuhan observed no significant effect of O_3_ on mortality in CEVD [32]. However, the results were different in regions with low O_3_ pollution. In rural Australia, where O_3_ concentrations remained in the single digits year-round, the association between O_3_ concentrations and daily hospitalizations for CVD and CEVD was negative (IRR: 0.96, 95% CI: 0.91–1.01) [9]. Studies of the elderly in New England, also from low-pollution areas, have not observed a protective or detrimental effect of O_3_ on CVD mortality [33]. According to another study in the United Kingdom, O_3_ has shown a negative correlation with CVD mortality. The ozone concentration range in this area was 40.5–63 μg/m^3^ [13]. It is suggested that ozone pollution control may be effective in reducing mortality from CVD and CEVD. Previous studies have shown that exposure to high concentrations of ozone decreases the high-frequency band of human heart rate variability and triggers the release of biomarkers associated with coagulation, platelet dysfunction, and endothelial damage. As a result, it increases the risk of CVD. [34]. However, the adverse effects of O_3_ on CVD and CEVD, and the underlying mechanisms, are not yet fully understood. Further studies are needed to clarify these mechanisms.

We also found that the effect of O_3_ on mortality from CVD and CEVD was greater in the warm season compared to the cold season. After adjusting for temperature, humidity, and other pollutants, we found that for each 10 μg/m^3^ increase in ozone concentration during the warm season, the mortality risk from CVD and CEVD in the elderly increased by 1.6% (95% CI: 0.7–2.5%). The relationship between ozone and cardiovascular disease in the elderly was not observed during the cold season. Previous studies have reached similar conclusions. A study in Lanzhou, China, showed that the effect of O_3_ on CVD mortality was stronger in the warm season than in the cold season [35]. Additionally, research in northern China has demonstrated that the association between O_3_ and all-cause mortality is stronger during the warm season [27]. This is likely because O_3_ is a secondary pollutant that is highly influenced by temperature, humidity, wind, and other environmental factors. Higher temperatures can promote ozone production, especially in northern, eastern, and northeastern Chinese cities where solar temperatures are higher during the warm season [36]. These findings may support our conclusions. A randomized crossover study of healthy young volunteers may provide a biological basis for the relationship between heat and ozone. The results suggest that the systemic effects of O_3_ exposure vary with temperature. O_3_ may activate the fibrinolytic pathway at appropriate temperatures. However, ozone may impair the fibrinolytic pathway which may prevent the growth of blood clots and lead to thrombosis at high temperatures [37]. The physiological mechanisms by which ozone and high temperatures interact to affect cardiovascular disease are not yet well elucidated. This may involve multiple pathways, and further studies are needed to elucidate these mechanisms.

We also found that the RR values were highest at a 1-day lag throughout the year, as well as during the warm season. The RR was 1.002 (95% CI: 1.000–1.004) and 1.005 (95% CI: 1.002–1.007) for each 10 μg/m^3^ increase in ozone concentration. The cumulative RR was maximum at a six-day lag (lag06). The cumulative RR for the whole year was 1.009 (95% CI: 1.001–1.016). The cumulative RR for the warm season was 1.017 (95% CI: 1.009–1.026). These findings align with results from Nanchang. The highest risk of death was observed at a 1-day lag. For each 10 μg/m^3^ rise in ozone concentration, the risk of death from CVD increased by 1.26% (0.68–1.8%). The highest cumulative multi-day lag effect was observed at a 0–6 days lag [8]. In Guangzhou, the highest CVD mortality risk was also observed at a 1-day lag. Differently, the cumulative effect was highest at 0–3 days [38]. The delayed impact of ozone on public health may be influenced by several factors, including the population structure, level of economic development, population-weighted ozone concentrations, regional public health policies, and healthcare resource allocation. The significant heterogeneity suggests that it is important to investigate and tailor public health policies to specific regions. Notably, previous studies have shown that ozone pollution is not a risk factor for CVD and CEVD in areas with sufficiently low ozone concentrations. Therefore, developing region-specific public health strategies, based on research, along with effective air pollution control policies, can significantly reduce CVD and CEVD mortality.

### 4.2. Subgroups More Sensitive to O_3_

Further subgroup analyses were conducted to examine the sensitivity of different population groups to O_3_ exposure. The results indicated that males were more susceptible to ozone, with statistically significant findings. However, females exhibited a more immediate response to O_3_ pollution. We found that males had the highest cumulative RR at lag06 and females had the highest cumulative RR at lag03. In contrast to our findings, some studies have shown that females are more sensitive to ozone exposure [8,26,30]. These studies concluded that females are more susceptible to air pollutants because their airways are shorter and they breathe differently than males. However, studies focusing on populations older than 65 years have reported results consistent with our findings [28,39,40]. A possible explanation for our findings could be that males tend to work or be more active outdoors, leading to greater exposure to air pollution [41]. Additionally, males may be more likely to pick up bad habits such as smoking and drinking. In China, the prevalence of smoking is 50.5% for males and 2.1% for females. It has been demonstrated that smokers are more susceptible to short-term air pollution exposure than never-smokers [42]. A nationwide study in China showed that individuals with healthier lifestyles had a reduced risk of cardiovascular disease, with reductions of approximately 38% and 62% under moderate and good lifestyle conditions, respectively, compared to poor lifestyles [43]. This suggests that adopting a healthy lifestyle can minimize the risk of CVD and CEVD.

We did not find any differences in the susceptibility to ozone pollution between populations with varying levels of education. In contrast to our study, previous research typically indicates that individuals with lower levels of education are more susceptible to the effects of ozone. A study conducted across 272 cities in China showed that for every 10 μg/m^3^ increase in ozone concentration, total mortality was four times higher in the lower-educated population compared to the higher-educated population [27]. Educational level has been proven a significant factor in the health effects of air pollution [44]. It is speculated that lower levels of healthcare awareness and limited financial resources in less educated groups contribute to poorer health outcomes [45]. Moreover, the low educated population is usually engaged in outdoor or manual labor which leads to greater exposure to ozone pollution. The difference between this study and previous research may be attributed to the fact that this study primarily focused on elderly individuals aged over 65, with a majority of participants having an education level of junior high school or below. Additionally, there was a large disparity in the sample sizes between the two groups.

This study found that unmarried, widowed, and divorced individuals are more sensitive to ozone pollution compared to married people. Research on the effects of air pollution by marital status is limited. It is possible that married individuals are healthier and less prone to depressive symptoms compared to individuals with unconventional marital status. Married individuals typically have access to emotional support and practical assistance from their partners, which enhances their ability to cope with environmental stressors. In contrast, unmarried, widowed, or divorced individuals may face more isolated social relationships, lacking sufficient support, and are more vulnerable to the negative effects of external pressures. Previous studies have shown that married individuals generally have better health outcomes and are less likely to experience depression. In contrast, unmarried, widowed, or divorced individuals often face a higher risk of depression, and depressive symptoms are closely associated with the onset of cardiovascular and cerebrovascular diseases [46]. Furthermore, certain depressive symptoms, including sleep disturbances and loneliness, are strongly correlated with the occurrence of CVD and CEVD [47].

We also found that outdoor workers were more vulnerable to O_3_ exposure than indoor workers. In particular, the negative impact of ozone on outdoor workers was already statistically significant at lag0. It is clear that outdoor workers are more exposed and vulnerable to ozone pollution. In addition, low-income individuals and farmers, who make up the majority of outdoor workers, are at higher risk. Studies have shown that low-income individuals are more vulnerable to ill health than high-income individuals [48] and at higher risk of death from exposure to air pollution [49]. CVD may occur within hours of exposure to ozone pollution [50]. Consequently, a negative effect of ozone on CVD and CEVD mortality was observed even at lag day 0. These subgroup analyses identified populations more sensitive to ozone pollution. They provide new and more targeted perspectives for future strategies to manage the burden of cardiovascular disease in Shenyang. However, more in-depth studies on socio-economic status (income, education, and occupation) and marital status are needed in the future.

### 4.3. Synergy EFfect Between PM_2.5_ and O_3_

Our study found a synergistic effect between ozone and PM_2.5_. Coexisting high concentrations of PM_2.5_ and O_3_ increase the mortality effect of CVD and CEVD. Studies conducted in Chengdu, China, and nationwide have reported similar findings, demonstrating significant synergistic effects of ozone and PM_2.5_ on CVD mortality in the population [51,52]. Biological mechanisms have been proposed to explain the potential interaction between PM_2.5_ and O_3_ pollution on cardiovascular mortality, despite the lack of clear evidence of a direct synergistic effect of these two pollutants on CVD and CEVD. Toxicological experiments conducted in rats have demonstrated that particulate matter can act as a carrier of O_3_ and deliver O_3_ into body [53]. Furthermore, studies have suggested that PM_2.5_ and O_3_ act on the human body with similar pathological pathways, such as inflammatory responses and oxidative stress, which may lead to interactions between the two pollutants [54]. This evidence hints at the existence of synergies between PM_2.5_ and O_3_, warranting further investigation and the development of policies for the co-control of both pollutants.

### 4.4. Limitations

An implication of this study is to recommend that individuals suffering from CVD and CEVD avoid exposure to higher levels of ambient PM_2.5_ and O_3_ in order to protect their health.

However, several limitations exist in our study. Firstly, previous studies on CEVD are limited, which we did not analyze meticulously and thoroughly. Secondly, the pollutant concentrations in this study were obtained from fixed-site detectors, which do not provide a completely accurate picture of the actual exposure of individuals and may differ from the real exposure of the population to ozone pollution. In future research, we plan to conduct studies at the individual level, using personal samplers to record air pollution exposure. Additionally, we will collect data on participants’ specific lifestyle and the time spent indoors and outdoors. Thirdly, the population data collected in this study was not sufficiently comprehensive. Future surveys should include factors such as smoking status, alcohol consumption, BMI, and living area. Finally, this study only examined the synergistic effect between O_3_ and PM_2.5_. Further research is needed to explore the synergistic effects of O_3_ with other pollutants or meteorological factors.

## 5. Conclusions

This study found that ozone pollution is associated with an increased risk of mortality from CVD and CEVD in the elderly, with this effect being more pronounced during warmer seasons and exhibiting a lag effect. The study also identified that, among individuals aged 65, males, those with non-traditional marital statuses, and outdoor workers are more susceptible to the health impacts of ozone pollution. No significant differences in susceptibility to ozone were found across different educational levels. Furthermore, this study revealed a synergistic effect between ozone and PM_2.5_. The combined impact of these pollutants on public health may be much greater than that of each pollutant individually. These findings contribute to a more comprehensive understanding of the health impacts of ozone and PM_2.5_. These findings, combined with the existing evidence, provide better estimates of the future burden of disease. They can help policymakers develop more targeted environmental policies that are effective in improving public health outcomes.

## Figures and Tables

**Table 1:** Basic characteristics of the participants. Data are mean ± standard deviation or n(%).

Characteristics		Overall (n = 117,604)
	Age	80.3 ± 7.85
**Gender**	Male	59,226 (50.4)
	Female	58,373 (49.6)
**Level of education**	Junior high school and below	102,232 (86.9)
	High school and above	15,372 (13.1)
**Marital status**	Married	61,283 (52.1)
	Single, divorced and widowed	55,096 (46.8)
	Unknow	1225 (1.0)
**Occupation**	Indoor	56,469 (48.0)
	Outdoor	46,632 (39.6)
	Unknow	14,503 (12.3)
**Cause of deaths**	CVD (I05~I52)	72,724 (61.8)
	CEVD (I60~I69)	44,880 (38.2)

**Table 2:** Descriptive statistic of O_3_, PM_2.5_, SO_2_, NO_2,_ temperature and relative humidity from 2014 to 2018.

	Mean	SD	Min	Max	P25	P50	P75
**O_3_ (μg/m^3^)** O_3_-warm O_3_-cold	66.7 84.7 41.9	40.3 40.5 22.2	9 10 9	250 250 140	35 56 24	59 79 37	88 107 54
**PM_2.5_ (μg/m^3^)** PM_2.5_-warm PM_2.5_-cold	56.5 41.6 77.6	47.3 31.7 56.7	4 4 10	848 632 848	28 24 41	43 35 65	72 51 101
**SO_2_ (μg/m^3^)** SO_2_-warm SO_2_-cold	47.9 19.8 87.6	55.1 12.0 66.6	3 3 10	527 120 527	15 11 39	27 17 70	57 26 114
**NO_2_ (μg/m^3^)** NO_2_-warm NO_2_-cold	41.8 36.3 49.7	17.1 14.0 18.0	12 12 14	125 109 125	29 27 36	39 34 48	51 43 61
**Temperature (°C)** Temperature-warm Temperature-cold	9.1 18.5 −4.2	13.1 6.7 7.2	−23 −5 −23	32 32 16	−3 14 −9	11 20 −5	21.2 24 1
**RH (%)** RH-warm RH-cold	59.9 62.9 55.6	16.3 17.0 14.2	15 15 22	98 98 91	47.8 52 45	61 66 55	72 75 66

**Table 3:** Spearman’s rank correlation coefficients between pollutants and meteorological variables (* *p* < 0.01).

	PM_2.5_	PM_10_	SO_2_	NO_2_	CO	O_3_-8 h	T	Rh
Rh (%)	−0.04	−0.10 *	−0.22	−0.03	0.16 *	−0.08 *	0.32 *	1
T (°C)	−0.39 *	−0.28 *	−0.068 *	−0.41 *	−0.22 *	0.69 *	1	
O_3_-8 h (μg/m^3^)	−0.16 *	−0.06 *	−0.39 *	−0.35 *	−0.2 *	1		
CO (mg/m^3^)	0.8 *	0.65 *	0.63 *	0.73 *	1			
NO_2_ (μg/m^3^)	0.74 *	0.63 *	0.71 *	1				
SO_2_ (μg/m^3^)	0.71 *	0.52 *	1					
PM_10_ (μg/m^3^)	0.89 *	1						
PM_2.5_ (μg/m^3^)	1							

**Table 4:** The relative risk (RR) of CEVD and CVD mortality associated with ambient O_3_ concentration (per 10 μg/m^3^).

	O_3_ (RR)	Warm Season O_3_ (RR)	Cold Season O_3_ (RR)
**Model 1**	**1.027 (1.019~1.034)**	**1.032 (1.024~1.040)**	1.005 (0.988~1.023)
**Model 2**	**1.008 (1.000~1.016)**	**1.016 (1.007~1.025)**	1.015 (0.993~1.037)
**Model 3**	1.007 (0.999~1.015)	**1.016 (1.007~1.025)**	1.016 (0.993~1.038)
**Model 4**	**1.007 (1.000~1.015)**	**1.016 (1.007~1.025)**	1.007 (0.986~1.029)

**Table 5:** The relative risk (lag0–7) and the cumulative relative risk (lag00–07) per 10 μg/m^3^ of O_3_ elevation for lag 0–7 days.

Lag	O_3_	Warm Season O_3_	Cold Season O_3_
lag0	1.002 (0.998–1.006)	1.002 (0.997–1.006)	1.003 (0.994–1.012)
lag1	**1.005 (1.002–1.007)**	**1.007 (1.004–1.010)**	1.003 (0.997–1.010)
lag2	**1.002 (1.000–1.004)**	**1.005 (1.002–1.007)**	0.999 (0.994–1.005)
lag3	0.999 (0.997–1.001)	1.001 (0.999–1.004)	0.996 (0.991–1.001)
lag4	0.998 (0.997–1.000)	1.000 (0.998–1.002)	0.997 (0.992–1.002)
lag5	1.001 (0.999–1.002)	**1.000 (1.000–1.003)**	1.001 (0.997–1.006)
lag6	**1.002 (1.000–1.005)**	**1.002 (1.000–1.005)**	1.005 (0.999–1.011)
lag7	0.998 (0.995–1.002)	0.998 (0.995–1.002)	1.002 (0.994–1.009)
lag00	1.002 (0.998–1.006)	1.002 (0.997–1.006)	1.003 (0.993–1.012)
lag01	**1.007 (1.002–1.011)**	**1.008 (1.003–1.014)**	1.006 (0.994–1.018)
lag02	**1.009 (1.003–1.014)**	**1.013 (1.007–1.019)**	1.005 (0.991–1.020)
lag03	**1.007 (1.001–1.013)**	**1.015 (1.008–1.021)**	1.001 (0.985–1.018)
lag04	**1.006 (1.000–1.013)**	**1.014 (1.007–1.022)**	0.998 (0.980–1.017)
lag05	**1.006 (1.000–1.014)**	**1.015 (1.007–1.023)**	1.000 (0.980–1.020)
lag06	**1.009 (1.001–1.016)**	**1.017 (1.009–1.026)**	1.005 (0.985–1.023)
lag07	**1.007 (1.000–1.015)**	**1.016 (1.007–1.025)**	1.007 (0.986–1.002)

**Table 6:** The RR of CEVD and CVD mortality for each 10 μg/m^3^ increase in O_3_ at different PM_2.5_ concentrations in warm season (lag = 1).

Model	Pollutants	RR (95% CI)
Single-pollutant analysis	O_3_ (PM_2.5low_)	0.998 (0.995–1.004)
	O_3_ (PM_2.5high_)	1.006 (1.000–1.007)
Two-pollutant analysis	O_3_-PM_2.5low_	0.998 (0.992–1.003)
	O_3_-PM_2.5high_	1.001 (1.000–1.004)

## Data Availability

The data presented in this study are available on request from the corresponding author.
